# Risk Factors and Mortality Among Women With Interval Breast Cancer vs Screen-Detected Breast Cancer

**DOI:** 10.1001/jamanetworkopen.2024.11927

**Published:** 2024-05-20

**Authors:** Huiyeon Song, Thi Xuan Mai Tran, Soyeoun Kim, Boyoung Park

**Affiliations:** 1 Department of Preventive Medicine, Hanyang University College of Medicine, Seoul, Republic of Korea; 2 Biomedical Research Institute, Seoul National University Hospital, Seoul, Republic of Korea; 3 Hanyang Institute of Bioscience and Biotechnology, Hanyang University, Seoul, Republic of Korea

## Abstract

**Importance:**

The risk factors for interval breast cancer (IBC) compared with those for screen-detected breast cancer (SBC) and their association with mortality outcomes have not yet been evaluated among Korean women.

**Objective:**

To evaluate risk factors associated with IBC and survival among Korean women with IBC compared with those with SBC.

**Design, Setting, and Participants:**

This retrospective cohort study used data from the Korean National Health Insurance Service Database. Women who participated in a national mammographic breast cancer screening program between January 1, 2009, and December 31, 2012, were included. Mortality outcomes were calculated from the date of breast cancer diagnosis to the date of death or December 31, 2020. Data were analyzed from March 1 to June 30, 2023.

**Exposure:**

Breast cancer diagnosed within 6 to 24 months after a negative screening result (ie, IBC) or within 6 months after a positive screening result (ie, SBC).

**Main Outcomes and Measures:**

Risk factors and survival rates for IBC and SBC.

**Results:**

This study included 8702 women with IBC (mean [SD] age, 53.3 [8.6] years) and 9492 women with SBC (mean [SD] age, 54.1 [9.0] years). Compared with SBC, the probability of IBC decreased as mammographic density increased. Lower body mass index, menopausal status, hormone replacement therapy (HRT) use, and lack of family history of breast cancer were associated with a higher likelihood of IBC. When stratified by detection time, younger age at breast cancer diagnosis and family history of breast cancer were associated with an increased likelihood of IBC diagnosed at 6 to 12 months but a decreased likelihood of IBC diagnosed at 12 to 24 months. Overall mortality of IBC was comparable with SBC, but total mortality and cancer-related mortality of IBC diagnosed between 6 and 12 months was higher than that of SBC.

**Conclusions and Relevance:**

The findings of this cohort study suggest that breast density, obesity, and HRT use were associated with IBC compared with SBC. These findings also suggest that higher supplemental breast ultrasound use among Korean women, especially those with dense breasts, could be attributed to a lower incidence of IBC among women with dense breasts compared with women with SBC, due to greater detection. Finally, overall mortality of IBC was comparable with that of SBC.

## Key Points

**Question:**

What are the established risk factors for women with interval breast cancer (IBC) vs screen-detected breast cancer (SBC), and how might they be associated with mortality outcomes?

**Findings:**

In this cohort study of 18 194 Korean women who underwent mammographic breast cancer screening, breast density, obesity, and hormone replacement therapy use were associated with IBC compared with SBC. Overall mortality of IBC was comparable with that of SBC.

**Meaning:**

These findings suggest that breast density is associated with IBC; additional research is warranted to determine whether a shortened screening period and new screening strategies are needed for women with risk factors for IBC.

## Teaser

This cohort study of Korean women uses mammographic breast cancer screening data to assess the risk factors for interval breast cancer vs screen-detected breast cancer and investigates the associated mortality outcomes.

## Introduction

According to GLOBOCAN estimates, female breast cancer was the most common incident cancer type in 2020, with approximately 2.3 million new cases, accounting for 24.5% of all cancer diagnoses.^1^ In Korea, breast cancer is the most common cancer type in women aged 35 to 64 years, and its incidence has been increasing since nationwide cancer statistics were first reported in 1999.^2^ To reduce the burden of breast cancer through early detection and treatment, mammographic breast cancer screening is recommended for average-risk women in many countries.^3^ In Korea, mammography is provided biennially to women aged 40 years or older as a nationwide organized screening program.^4^

Breast cancer diagnosed after a negative screening but before the next scheduled mammogram is referred to as interval breast cancer (IBC).^5,6^ There are 2 types: (1) “true” IBC, in which cancer did not present on the previous mammogram and thus developed during the screening interval; and (2) tumors missed on the previous mammogram (ie, false-negative results).^5^ Compared with screen-detected breast cancer (SBC), IBC has more aggressive characteristics and a worse prognosis.^5^ Previous studies have identified the following risk factors for IBC: high breast density, current hormone replacement therapy (HRT) use, young age, premenopausal status, and family history of breast cancer.^7^ Previous studies focused on IBC risk factors and characteristics have been conducted mainly in Western countries, where early mammographic breast cancer screening is recommended. However, little is known about the risk factors for IBC among Asian women.

Identification of epidemiologically associated risk factors for IBC and survival rates of Asian women with IBC compared with those with SBC would be useful for improving screening strategies for women in Asia. Therefore, this study aimed to compare established risk factors for IBC and SBC among Korean women using data from a nationwide mammographic screening program. Furthermore, mortality outcomes of IBC were compared with those of SBC.

## Methods

The institutional review board of Hanyang University College of Medicine, Republic of Korea, approved the protocol for this cohort study. In addition, the National Health Insurance Sharing Service System (NHIS) approved the use of the NHIS database, which was constructed after individual identities were anonymized. The need for informed consent was thus waived due to secondary data analysis. The study followed the Strengthening the Reporting of Observational Studies in Epidemiology (STROBE) reporting guideline.

### Study Design and Population

This study used data from the National Health Insurance Service National Health Information Database (NHIS-NHID). As a single compulsory health insurance system in Korea, the NHIS-NHID includes information on demographics, health care use, vital statistics, and national health screening results for the entire Korean population.^8^

This study included data from all women who underwent national mammographic breast cancer screening between January 1, 2009, and December 31, 2012 (Figure). The mammographic screening film was read by a trained radiologist and the results were recorded according to the Breast Imaging Reporting and Data System, Fourth Edition (BI-RADS 4). A positive screening result was defined as a BI-RADS final assessment of 0, 4, or 5 (incomplete, suspicious abnormality, or highly suggestive of malignancy), and negative screening results were defined as 1, 2, or 3 (negative, benign finding, or probably benign) based on the American College of Radiology guideline.^9^ Participants were excluded if they met the following criteria: (1) were younger than 40 years or aged 75 years or older at the time of screening (n = 347 845), according to the recommended age in Korean guidelines for breast cancer screening^10^; (2) had a history of breast cancer before the mammographic screening date (n = 27); (3) were missing information on breast cancer screening results (n = 45); (4) were missing information on mammographic breast density (n = 147 259); or (5) had received any type of cancer diagnosis before screening (n = 179 128). Of the 6 772 811 women screened, 5 628 035 had negative results and 1 144 776 had positive results.

**Figure.**
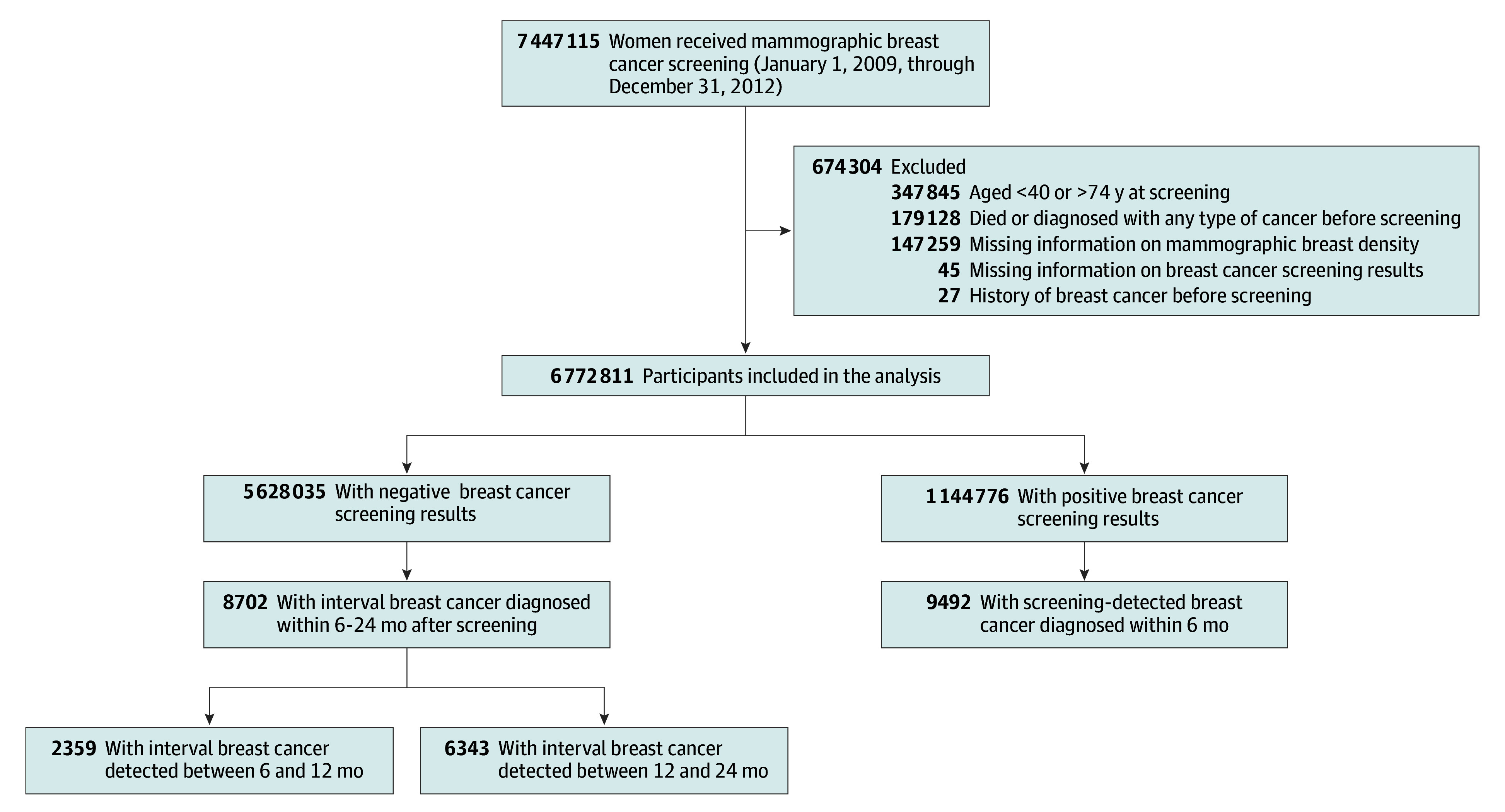
Study Flow Diagram

### Definition of IBC and SBC

Breast cancer was defined as any breast cancer event according to *International Classification of Diseases, Tenth Edition* (*ICD-10*) codes for invasive breast cancer (C50.0-C50.9) and ductal carcinoma in situ (D05.0-D05.9) and as any cancer event registered with the rare incurable disease code for cancer. If a breast cancer diagnosis occurred within 6 months from the date of mammographic screening among women with positive screening results, the case was defined as SBC, considering the time of referral to a hospital for further examination and pathologic confirmation. The 6-month period to define SBC was applied in another claim-based study in Korea.^11^ Among women with negative screening results, cancers diagnosed within the first 6 months after negative screening were considered false-negative results and were excluded. Considering the definition of IBC and the screening cycle of other studies,^12,13,14^ the case was defined as IBC if the diagnosis of breast cancer occurred during the period 6 to 24 months after negative breast cancer screening. Considering the recommendation of annual breast cancer screening in other guidelines,^3^ IBC was further classified as breast cancer diagnosed either 6 to 12 months or 12 to 24 months after negative screening. Breast cancer was defined as a combination of the *ICD-10* codes for invasive breast cancer and ductal carcinoma in situ and the special code for cancer.^15^ Based on this definition, we identified 8702 women with IBC (2359 were diagnosed between 6 and 12 months after negative screening, and 6343 were diagnosed between 12 and 24 months) from 5 628 035 with negative results (0.15%) and 9492 women with SBC from 1 144 776 with positive results (0.83%).

### Established Risk Factors for Breast Cancer Considered in This Study

During cancer screening, participants were asked to complete a self-administered questionnaire about their health behaviors and risk factors for breast cancer. The following risk factors were considered in this study: age at diagnosis (40-49, 50-59, or 60-74 years), mammographic density according to BI-RADS 4 category (1, <25% glandular; 2, 25%-50% glandular; 3, 51%-75% glandular; or 4, >75% glandular), body mass index (BMI [calculated as weight in kilograms divided by height in meters squared]; underweight, <18.5; normal, 18.5 to <23.0; overweight, 23.0 to <25.0; or obesity, ≥25.0), age at menarche (<15, 15-16, or ≥17 years), menopausal status (premenopausal or postmenopausal), oral contraceptive use (never or ever), HRT use after menopause (never or ever), parity (nulliparous, primiparous, or multiparous), breastfeeding experience (never or ever), family history of breast cancer in first-degree relatives (no or yes), current alcohol consumption (no or yes [<1 or ≥1 per week during the last year]), and physical activity (no or yes [<1 or ≥1 time per week at time of screening]).

### Mortality

Mortality and causes of death of individuals between 2009 and 2020 were confirmed by linking the NHIS-NHID data with mortality data from the Korea National Statistical Office (KNSO) using a unique 13-digit resident registration number. The KNSO death certificate includes the cause of death according to the *ICD-10 *code and the date of death. First, all-cause mortality, defined as death from any cause, was assessed. Causes of death were classified as breast cancer–related death (recorded cause of death as breast cancer) or deaths other than breast cancer (all other causes). Moreover, causes of death were also classified into cancer-related deaths (recorded causes of death as any cancer) and deaths other than cancer (all other causes).

### Statistical Analysis

Descriptive statistics on breast cancer risk factors among women with SBC or IBC were compared using the *t* test or χ^2^ test. Continuous variables are presented as means with SDs, and categorical variables are presented as numbers with percentages. Logistic regression with the aforementioned established risk factors for breast cancer was used to identify epidemiologically associated factors for IBC compared with SBC. Odds ratios (ORs) and 95% CIs for each factor with and without adjustments for other factors are presented. Analysis was performed for all participants with IBC and stratified by IBC diagnosed between 6 and 12 months and between 12 and 24 months. Given that mammographic density could affect IBC itself and its association with other factors,^16^ a multiple logistic regression analysis of the association between established risk factors for breast cancer and IBC compared with SBC was performed, stratified by breast density (nondense [BI-RADS density categories 1-2] and dense [BI-RADS density categories 3-4]).

Mortality outcomes for IBC compared with SBC were analyzed using Cox proportional hazards regression analysis. Follow-up for the mortality outcome was calculated from the date of breast cancer diagnosis to the date of death or December 31, 2020. Hazard ratios (HRs) for mortality risk are presented as unadjusted, adjusted for age at diagnosis, and adjusted for all of the aforementioned known risk factors for breast cancer for all-cause mortality, breast cancer–related death, cancer-related death, deaths other than breast cancer, and deaths other than cancer. A 2-sided *P* < .05 was considered statistically significant. Statistical analysis was performed with SAS, version 9.4 (SAS Institute). Data were analyzed from March 1 to June 30, 2023.

## Results

### Characteristics of Women With IBC vs SBC

A total of 18 194 women were included in the study; 9492 had SBC and 8702 had IBC (Table 1). Of the 8702 women with IBC, 2359 (27.1%) were diagnosed between 6 and 12 months after negative breast cancer screening and 6343 (72.9%) were diagnosed between 12 and 24 months. Women with IBC were younger at the time of cancer diagnosis (mean [SD] age, 53.3 [8.6] years) compared with those with SBC (mean [SD] age, 54.1 [9.0] years). The proportion of BI-RADS category 4 tumors was higher for women with IBC (2031 [23.3%]) compared with those with SBC (1895 [20.0%]). Compared with women with SBC, a lower proportion of women with IBC had obesity (2590 [29.8%] vs 3328 [35.1%]) and a higher proportion of women with IBC used HRT (768 [18.2%] vs 750 [14.3%]). Proportions of younger women, women with dense breasts, premenopausal women, and women with a family history of breast cancer were lower among women with IBC diagnosed at 12 to 24 months compared with at 6 to 12 months.

**Table 1.** Characteristics of the IBC and SBC Groups^a^

Risk factor	Cancer type			IBC diagnostic period^b^		
	SBC (n = 9492)	IBC (n = 8702)	*P* value^c^	6-12 mo (n = 2359)	12-24 mo (n = 6343)	*P* value^c^
Age at diagnosis, y						
Mean (SD)	54.1 (9.0)	53.3 (8.6)	<.001	52.2 (8.4)	53.7 (8.7)	.13
40-49	3282 (34.6)	3342 (38.4)	<.001	1058 (44.9)	2284 (36.0)	<.001
50-59	3558 (37.5)	3303 (38.0)		856 (36.3)	2447 (38.6)	
60-74	2652 (27.9)	2057 (23.6)		445 (18.9)	1612 (25.4)	
BI-RADS breast density category^d^						
1	1222 (12.9)	1231 (14.2)	<.001	299 (12.7)	932 (14.7)	<.001
2	2554 (26.9)	2120 (24.4)		521 (22.1)	1599 (25.2)	
3	3821 (40.3)	3320 (38.2)		943 (40.0)	2377 (37.5)	
4	1895 (20.0)	2031 (23.3)		596 (25.3)	1435 (22.6)	
BMI						
Underweight (<18.5)	187 (2.0)	213 (2.5)	<.001	68 (2.9)	145 (2.3)	.08
Normal (18.5-23)	3648 (38.4)	3702 (42.5)		1032 (43.8)	2670 (42.1)	
Overweight (23-25)	2328 (24.5)	2196 (25.2)		597 (25.3)	1599 (25.2)	
Obesity (≥25)	3328 (35.1)	2590 (29.8)		662 (28.1)	1928 (30.4)	
Missing	1 (0)	1 (0)		0	1 (0)	
Age at menarche, y						
<15	2798 (29.5)	2774 (31.9)	<.001	773 (32.8)	2001 (31.6)	.65
15-16	4018 (42.3)	3690 (42.4)		989 (41.9)	2701 (42.6)	
≥17	2435 (25.7)	2039 (23.4)		548 (23.2)	1491 (23.5)	
Missing	241 (2.5)	199 (2.3)		49 (2.1)	150 (2.4)	
Menopausal status						
Premenopause	4123 (43.4)	4390 (50.5)	<.001	1250 (53.0)	3140 (49.5)	.01
Postmenopause	5256 (55.4)	4212 (48.4)		1086 (46.0)	3126 (49.3)	
Missing	113 (1.2)	100 (1.2)		23 (1.0)	77 (1.2)	
Oral contraceptive use						
Never	7502 (79.0)	6983 (80.3)	.12	1901 (80.6)	5082 (80.1)	.85
Ever	1373 (14.5)	1195 (13.7)		321 (13.6)	874 (13.8)	
Missing	617 (6.5)	524 (6.0)		137 (5.8)	387 (6.1)	
HRT use among postmenopausal women						
Never	3570 (67.9)	2637 (62.6)	<.001	684 (63.0)	1953 (62.5)	.95
Ever	750 (14.3)	768 (18.2)		197 (18.1)	571 (18.3)	
Missing	936 (17.8)	807 (19.2)		205 (18.9)	602 (19.3)	
Parity						
Nulliparous	634 (6.7)	542 (6.2)	.41	159 (6.7)	383 (6.0)	.005
Primiparous	1389 (14.6)	1228 (14.1)		354 (15.0)	874 (13.8)	
Multiparous	7354 (77.5)	6828 (78.5)		1821 (77.2)	5007 (78.9)	
Missing	115 (1.2)	104 (1.2)		25 (1.1)	79 (1.3)	
Breastfeeding experience						
Never	1598 (16.8)	1669 (19.2)	<.001	484 (20.5)	1185 (18.7)	.13
Ever	7611 (80.2)	6792 (78.1)		1815 (76.9)	4977 (78.5)	
Missing	283 (3.0)	241 (2.8)		60 (2.5)	181 (2.9)	
Family history of breast cancer						
No	8790 (92.6)	8136 (93.5)	.02	2157 (91.4)	5979 (94.3)	<.001
Yes	702 (7.4)	566 (6.5)		202 (8.6)	364 (5.7)	
Current alcohol consumption						
No	7473 (78.7)	6639 (76.3)	<.001	1779 (75.4)	4860 (76.6)	.31
Yes	1972 (20.8)	2021 (23.2)		571 (24.2)	1450 (22.9)	
Missing	47 (0.5)	42 (0.5)		9 (0.4)	33 (0.5)	
Physical activity						
No	2563 (27.0)	2275 (26.1)	.27	579 (24.5)	1696 (26.7)	.12
Yes	6868 (72.4)	6380 (73.3)		1767 (74.9)	4613 (72.7)	
Missing	61 (0.6)	47 (0.5)		13 (0.6)	34 (0.5)	

Abbreviations: BI-RADS, Breast Imaging Reporting and Data System; BMI, body mass index (calculated as weight in kilograms divided by height in meters squared); HRT, hormone replacement therapy; IBC, interval breast cancer; SBC, screen-detected breast cancer.

^a^ Unless indicated otherwise, values are presented as No. (%) of patients.

^b^ Subdivided by diagnosis time: within 6 and 12 months and 12 and 24 months.

^c^ χ^2^ test for categorical variables and *t* test for continuous variables.

^d^ Breast density category 1 was almost entirely fat (<25% glandular), category 2 indicated scattered fibroglandular density (25%-50% glandular), category 3 was heterogeneously dense (51%-75% glandular), and category 4 was extremely dense (>75% glandular).

### Risk Factors for IBC vs SBC

Table 2 presents established risk factors for breast cancer that were differentially associated with IBC relative to SBC. We observed that compared with SBC, as the BI-RADS mammographic density category increased, the probability of IBC decreased in women with density category 2 (adjusted OR [AOR], 0.76 [95% CI, 0.69-0.84]), category 3 (AOR, 0.70 [95% CI, 0.64-0.78]), and category 4 (AOR, 0.80 [95% CI, 0.72-0.90]) vs density category 1. Compared with women with obesity, lower BMI was associated with a higher likelihood of IBC among women with overweight (AOR, 1.19 [95% CI, 1.10-1.29]), normal weight (AOR, 1.24 [95% CI, 1.16-1.34]), and underweight (AOR, 1.36 [95% CI, 1.10-1.67]). For menopause and ever use of HRT after menopause, AORs of 1.21 (95% CI, 1.07-1.35) and 1.40 (95% CI, 1.25-1.57) were observed for IBC vs SBC. For family history of breast cancer and alcohol consumption, AORs of 0.89 (95% CI, 0.79-0.99) and 1.10 (95% CI, 1.02-1.18) were observed for IBC vs SBC.

**Table 2.** Risk Factors for IBC vs SBC

Risk factor	IBC vs SBC (n = 8702)		IBC at 6-12 mo vs SBC (n = 2359)^a^		IBC at 12-24 mo vs SBC (n = 6343)^a^	
	COR (95% CI)	AOR (95% CI)^b^	COR (95% CI)	AOR (95% CI)^b^	COR (95% CI)	AOR (95% CI)^b^
Age at diagnosis, y						
40-49	1.31 (1.22-1.42)	1.00 (0.89-1.12)	1.92 (1.70-2.17)	1.76 (1.48-2.10)	1.15 (1.06-1.24)	0.81 (0.72-0.92)
50-59	1.20 (1.11-1.29)	1.11 (1.02-1.20)	1.43 (1.27-1.63)	1.42 (1.24-1.63)	1.13 (1.04-1.23)	1.02 (0.93-1.11)
60-74	1 [Reference]	1 [Reference]	1 [Reference]	1 [Reference]	1 [Reference]	1 [Reference]
BI-RADS breast density^c^						
1	1 [Reference]	1 [Reference]	1 [Reference]	1 [Reference]	1 [Reference]	1 [Reference]
2	0.82 (0.75-0.91)	0.76 (0.69-0.84)	0.83 (0.71-0.98)	0.74 (0.63-0.87)	0.82 (0.74-0.91)	0.76 (0.69-0.85)
3	0.86 (0.79-0.95)	0.70 (0.64-0.78)	1.01 (0.87-1.17)	0.75 (0.64-0.87)	0.82 (0.74-0.90)	0.68 (0.61-0.76)
4	1.06 (0.96-1.18)	0.80 (0.72-0.90)	1.29 (1.10-1.50)	0.84 (0.71-1.01)	0.99 (0.89-1.11)	0.78 (0.69-0.89)
BMI						
Underweight (<18.5)	1.46 (1.20-1.79)	1.36 (1.10-1.67)	1.83 (1.37-2.44)	1.53 (1.14-2.06)	1.34 (1.07-1.68)	1.29 (1.02-1.62)
Normal (18.5-23)	1.30 (1.22-1.40)	1.24 (1.16-1.34)	1.42 (1.28-1.59)	1.25 (1.12-1.40)	1.26 (1.17-1.36)	1.24 (1.14-1.34)
Overweight (23-25)	1.21 (1.12-1.31)	1.19 (1.10-1.29)	1.29 (1.14-1.46)	1.23 (1.08-1.34)	1.19 (1.09-1.29)	1.18 (1.08-1.29)
Obesity (≥25)	1 [Reference]	1 [Reference]	1 [Reference]	1 [Reference]	1 [Reference]	1 [Reference]
Age at menarche, y						
<15	1.18 (1.09-1.28)	1.07 (0.98-1.16)	1.23 (1.09-1.39)	0.97 (0.85-1.10)	1.17 (1.07-1.27)	1.11 (1.01-1.22)
15-16	1.10 (1.02-1.18)	1.02 (0.95-1.10)	1.09 (0.97-1.23)	0.95 (0.84-1.07)	1.10 (1.01-1.19)	1.05 (0.97-1.14)
≥17	1 [Reference]	1 [Reference]	1 [Reference]	1 [Reference]	1 [Reference]	1 [Reference]
Menopausal status						
Premenopause	1.33 (1.25-1.41)	1.21 (1.07-1.35)	1.47 (1.34-1.61)	1.06 (0.88-1.27)	1.28 (1.20-1.37)	1.27 (1.12-1.44)
Postmenopause	1 [Reference]	1 [Reference]	1 [Reference]	1 [Reference]	1 [Reference]	1 [Reference]
Oral contraceptive use						
Never	1 [Reference]	1 [Reference]	1 [Reference]	1 [Reference]	1 [Reference]	1 [Reference]
Ever	0.94 (0.86-1.02)	0.93 (0.86-1.02)	0.92 (0.81-1.05)	0.94 (0.82-1.08)	0.94 (0.86-1.03)	0.93 (0.85-1.02)
HRT use among postmenopausal women						
Never	1 [Reference]	1 [Reference]	1 [Reference]	1 [Reference]	1 [Reference]	1 [Reference]
Ever	1.39 (1.24-1.55)	1.40 (1.25-1.57)	1.37 (1.15-1.64)	1.39 (1.16-1.66)	1.39 (1.23-1.57)	1.40 (1.24-1.58)
Parity						
Nulliparous	1 [Reference]	1 [Reference]	1 [Reference]	1 [Reference]	1 [Reference]	1 [Reference]
Primiparous	1.03 (0.91-1.19)	1.00 (0.86-1.17)	1.02 (0.82-1.25)	0.95 (0.76-1.20)	1.04 (0.89-1.21)	1.03 (0.87-1.21)
Multiparous	1.09 (0.96-1.22)	1.12 (0.97-1.28)	0.99 (0.82-1.18)	1.02 (0.83-1.25)	1.13 (0.99-1.29)	1.16 (0.99-1.35)
Breastfeeding experience						
Never	1.17 (1.09-1.26)	1.07 (0.99-1.16)	1.27 (1.13-1.42)	1.09 (0.97-1.23)	1.13 (1.04-1.23)	1.06 (0.97-1.16)
Ever	1 [Reference]	1 [Reference]	1 [Reference]	1 [Reference]	1 [Reference]	1 [Reference]
Family history of breast cancer						
No	1 [Reference]	1 [Reference]	1 [Reference]	1 [Reference]	1 [Reference]	1 [Reference]
Yes	0.87 (0.78-0.98)	0.89 (0.79-0.99)	1.17 (1.00-1.38)	1.18 (1.00-1.39)	0.76 (0.67-0.87)	0.77 (0.68-0.88)
Current alcohol consumption						
No	1 [Reference]	1 [Reference]	1 [Reference]	1 [Reference]	1 [Reference]	1 [Reference]
Yes	1.15 (1.08-1.24)	1.10 (1.02-1.18)	1.22 (1.09-1.35)	1.09 (0.98-1.22)	1.13 (1.05-1.22)	1.11 (1.02-1.20)
Physical activity						
No	1 [Reference]	1 [Reference]	1 [Reference]	1 [Reference]	1 [Reference]	1 [Reference]
Yes	1.05 (0.98-1.12)	1.02 (0.95-1.09)	1.14 (1.03-1.26)	1.10 (0.99-1.22)	1.02 (0.95-1.09)	0.99 (0.92-1.06)

Abbreviations: AOR, multivariate-adjusted odds ratio; BI-RADS, Breast Imaging Reporting and Data System; BMI, body mass index (calculated as weight in kilograms divided by height in meters squared); COR, crude odds ratio; HRT, hormone replacement therapy; IBC, interval breast cancer; SBC, screen-detected breast cancer.

^a^ Subdivided by diagnosis time: within 6 and 12 months and 12 and 24 months.

^b^ Multivariate logistic regression model adjusted for age at diagnosis, BI-RADS breast density, BMI, age at menarche, menopausal status, oral contraceptive use, HRT use among postmenopausal women, parity, breastfeeding experience, family history of breast cancer in first-degree relatives, alcohol consumption, and physical activity.

^c^ Breast density category 1 was almost entirely fat (<25% glandular), category 2 indicated scattered fibroglandular density (25%-50% glandular), category 3 was heterogeneously dense (51%-75% glandular), and category 4 was extremely dense (>75% glandular).

Decreased BI-RADS breast density category, lower BMI, and HRT use after menopause had an increased association with IBC compared with SBC, regardless of the diagnostic period after negative screening (6-12 and 12-24 months). However, age group and family history of breast cancer had different directions of association with IBC diagnostic period after negative screening. Younger age and family history of breast cancer were associated with an increased likelihood of IBC diagnosis at 6 to 12 months but with a decreased likelihood of IBC diagnosis at 12 to 24 months. When stratified by breast density (dense and nondense), associations of breast cancer risk factors with IBC, IBC diagnosed within 6 to 12 months, and IBC diagnosed within 12 to 24 months (eTables 1-3 in Supplement 1) were comparable with overall risk (Table 2).

### Mortality Risk Among Women With IBC vs SBC

The mean (SD) follow-up period after breast cancer diagnosis was 8.4 (1.9) years for women with IBC and 9.5 (2.1) years for those with SBC. Table 3 presents mortality outcomes among women with IBC compared with those with SBC. After adjusting for other covariates, no statistically significant increase or decrease in risk of death from all causes, breast cancer–related death, deaths other than breast cancer, or deaths other than cancer was observed for IBC compared with SBC. However, IBC had a 1.12-fold (95% CI, 1.01-1.24) higher risk of cancer-related death. When IBC was stratified by diagnostic period after a negative screening result, IBC diagnosed within 6 to 12 months had a 1.18-fold (95% CI, 1.02-1.37) higher risk of death from all causes, a 1.19-fold (95% CI, 1.01-1.41) higher risk of breast cancer–related death, and a 1.20-fold (95% CI, 1.03-1.40) increased risk of cancer-related deaths compared with SBC. Otherwise, increased mortality or cause-specific mortality was not observed for IBC at 12 to 24 months compared with SBC.

**Table 3.** Mortality Among Women With IBC vs SBC

Outcome	HR (95% CI)			
	SBC	IBC^a^	IBC at 6-12 mo	IBC at 12-24 mo
Death from all causes				
No.	NA	1356	941	1173
Model 1^b^	1 [Reference]	1.03 (0.94-1.14)	1.09 (0.94-1.26)	1.01 (0.91-1.13)
Model 2^c^	1 [Reference]	1.06 (0.96-1.16)	1.15 (0.99-1.33)	1.03 (0.92-1.14)
Model 3^d^	1 [Reference]	1.09 (0.99-1.20)	1.18 (1.02-1.37)	1.05 (0.94-1.17)
Breast cancer–related deaths				
No.	NA	929	648	793
Model 1	1 [Reference]	1.01 (0.90-1.13)	1.13 (0.96-1.34)	0.96 (0.85-1.09)
Model 2	1 [Reference]	1.02 (0.91-1.14)	1.15 (0.97-1.36)	0.97 (0.85-1.10)
Model 3	1 [Reference]	1.06 (0.94-1.19)	1.19 (1.01-1.41)	1.01 (0.89-1.15)
Deaths other than breast cancer				
No.	NA	427	293	380
Model 1	1 [Reference]	1.09 (0.90-1.32)	0.96 (0.71-1.30)	1.15 (0.93-1.41)
Model 2	1 [Reference]	1.17 (0.97-1.42)	1.31 (0.83-1.53)	1.19 (0.97-1.46)
Model 3	1 [Reference]	1.18 (0.98-1.43)	1.13 (0.83-1.53)	1.20 (0.97-1.48)
Cancer-related deaths				
No.	NA	1105	758	949
Model 1	1 [Reference]	1.06 (0.96-1.18)	1.13 (0.96-1.32)	1.03 (0.93-1.17)
Model 2	1 [Reference]	1.08 (0.97-1.20)	1.17 (1.00-1.36)	1.05 (0.93-1.17)
Model 3	1 [Reference]	1.12 (1.01-1.24)	1.20 (1.03-1.40)	1.09 (0.97-1.22)
Deaths other than cancer				
No.	NA	251	183	224
Model 1	1 [Reference]	0.84 (0.63-1.10)	0.87 (0.56-1.33)	0.82 (0.60-1.13)
Model 2	1 [Reference]	0.91 (0.69-1.20)	1.05 (0.68-1.61)	0.86 (0.63-1.17)
Model 3	1 [Reference]	0.91 (0.69-1.21)	1.06 (0.69-1.63)	0.86 (0.63-1.18)

Abbreviations: HR, hazard ratio; IBC, interval breast cancer; NA, not applicable; SBC, screen-detected breast cancer.

^a^ Subdivided into 2 groups according to diagnosis time: within 6 and 12 months and 12 and 24 months.

^b^ Model 1: IBC as the outcome without adjustment.

^c^ Model 2: IBC as the outcome, adjusted for age at diagnosis.

^d^ Model 3: IBC as the outcome, adjusted for age at diagnosis; Breast Imaging Reporting and Data System, Fourth Edition breast density; body mass index; age at menarche; menopausal status; oral contraceptive use; hormone replacement therapy experience; parity; breastfeeding experience; family history of breast cancer in first-degree relatives; alcohol consumption; and physical activity.

## Discussion

This study investigated differences in risk factors associated with IBC compared with those associated with SBC and mortality outcomes between IBC and SBC among Korean women who participated in a nationwide breast cancer screening program. In this study, increased breast density was associated with a lower likelihood of IBC; however, decreased BMI, HRT use after menopause, and alcohol consumption were associated with an increased likelihood of IBC compared with SBC. Age and family history of breast cancer had different associations according to the IBC diagnostic period after negative screening results. Younger age at the time of breast cancer diagnosis and family history of breast cancer were associated with an increased likelihood of IBC diagnosis 6 to 12 months after negative screening results but with a decreased likelihood of IBC diagnosis at 12 to 24 months.

The association between HRT use after menopause and increased risk of IBC has been well identified in previous studies.^7,16,17,18^ Studies have reported that rates of IBC increase with an increasing duration of HRT use, and the association between HRT use and IBC is greater than that between HRT and SBC.^19,20^ In previous studies, lower BMI was associated with a higher risk of IBC diagnosed within 12 months.^20,21,22^ Previous studies have reported a higher proportion of premenopausal women with IBC than women with SBC.^23,24^ These associations are consistent with the results of this study. The mechanisms of the previous studies underlying the associations among HRT use, lower BMI, premenopause, and IBC were mainly explained by the effect of these factors on dense breasts and the masking effect or independent increased risk of IBC due to higher breast density.^22,25^ However, associations between these factors and IBC compared with SBC after adjusting for breast density or stratified by breast density suggested independent associations with IBC.

A notable finding of this study was the inverse association between increased breast density and the risk of IBC. Previous studies have consistently reported that increased mammographic density in terms of BI-RADS categories, density percentage, or dense area was associated with an increased risk of IBC compared with SBC,^5,21,26^ which is contrary to the results of this study.^16^ Increased IBC risk among women with dense breasts or subtle false-negative findings on mammography may be attributed to the masking effect just described. One study suggested that only 42.5% of IBC cases were true IBC, 16.2% were false-negative results, and the remaining 41.2% included occult tumors or minimal signs or were unclassifiable.^17^ Another Korean study suggested that 32.5% of IBC cases could be classified as true IBC, 35% as false-negative results (missed cancers), and 32.5% as minimal signs.^26^ To reduce the possibility that missed cancer cases were included in IBC in this study, IBC was stratified by diagnostic period; however, the inverse association remained, contrary to previous studies.

Despite limited evidence of the benefits of supplemental breast ultrasound screening, breast ultrasonography in addition to mammography has made a substantial contribution to finding more breast cancer cases that would be missed by mammography alone in women with dense breasts.^27^ Another study showed similar IBC rates in women with dense breasts who underwent supplemental ultrasound screening as in women with nondense breasts.^28^ A clinical trial in Japan reported that the IBC rate was lower in women who underwent additional ultrasonography than in those who did not, regardless of breast density.^29^ Furthermore, in groups receiving supplemental ultrasonography, IBC rates were similar in women with dense and nondense breasts.^29^ Therefore, if women with dense breasts receive supplemental screening, IBC could be lower than in women with nondense breasts without supplemental examination. A nationwide survey in Korea reported that breast ultrasonography was performed extensively in women. A total of 42.1% of women aged 30 years or older received breast ultrasound and of those who received breast cancer screening, more than 50% had received breast ultrasound.^30^ In this study, we were unable to identify whether women in the high-density category received additional supplemental screening; however, considering that breast ultrasound is widely performed in Korea, it could be expected that a higher proportion of women with dense breasts would undergo additional breast ultrasound examinations, which presents an inverse association between the increase in density category and decrease in IBC. Thus, the contrasting results from previous studies might be attributed to health service use among Korean women and not to biological mechanisms.

An increased risk of IBC is associated with younger age compared with older age.^7^ Similarly, in our analysis stratified by diagnostic period (6-12 and 12-24 months), the crude OR suggested an increased risk of IBC in younger women than in older women. However, the multivariate analysis suggested that an increased risk of IBC was associated with younger age for IBC diagnosed at 6 to 12 months, whereas the opposite association was observed for IBC diagnosed at 12 to 24 months. Additionally, an opposite association was observed between the unadjusted OR and AOR in IBC diagnosed at 12 to 24 months compared with SBC, which was attributed to the confounding effect of menopausal status. Otherwise, premenopausal status, which had a significantly higher risk in the univariate analysis in both periods, showed only an increased association with IBC diagnosed at 12 to 24 months in the multivariate analysis. Owing to the close association between age and menopause, adjusting for one factor could affect the association of other factors with the risk of IBC.

In this study, a family history of breast cancer, which is known to be associated with an increased risk of IBC,^7^ had an increased association with IBC diagnosed at 6 to 12 months but a decreased association with IBC diagnosed at 12 to 24 months. In clinical guidelines, a more intensive screening is recommended at short intervals.^31,32^ These guidelines affect screening uptake behaviors, leading to increased IBC at 6 to 12 months and decreased IBC at 12 to 24 months.

In this study, we did not observe differences in overall mortality and cause-specific mortality between the SBC and IBC groups. However, IBC diagnosed 6 to 12 months after negative screening showed higher all-cause, breast cancer–related, and cancer-related mortality than SBC, suggesting the more aggressive nature of IBC diagnosed earlier after negative screening. Studies on the mortality of IBCs compared with SBCs have shown inconsistent results. For instance, IBCs are associated with a significantly higher unadjusted risk of breast cancer–related death than SBCs.^33^ In addition, IBCs have a significantly worse survival rate than SBCs in women with nondense breasts.^5^ The reasons for lower survival rates of IBC (<1 year) compared with SBC have been suggested to be greater tumor size, more aggressive nature (eg, more lymph node involvement), and a higher proportion of lobular histologic characteristics.^5,13,34,35^ However, recent studies have not reported differences in long-term survival between SBCs and IBCs.^36^ One study reported that except for the slightly larger size of IBC, the tumor characteristics of SBC and IBC were comparable.^37^ Because our population-based screening study could not compare the tumor characteristics of IBC and SBC, further hospital-based studies with available tumor information should be conducted.

### Strengths and Limitations

One strength of our study is that it was a large population-based study of women undergoing breast cancer screening. However, this study has some limitations. First, despite several previous studies defining IBC as cancer present after negative screening results and before the next scheduled screening,^7^ considering 20% of 25% of IBC cases are classified as false-negative results, we excluded breast cancers detected after less than 6 months of a negative result to exclude the possibility of missed cancer (false negative). However, considering that 10% to 30% of mammograms could have false-negative results due to technical or interpretive errors,^37,38,39,40,41^ some missed breast cancers could be included. Due to the unavailability of mammography films, we were unable to evaluate missed cancer or true IBC. In addition, we did not consider censoring the follow-up for breast cancer at the next screening, although screening intervals may be shorter for women with a family history of breast cancer. Therefore, screen-detected IBCs were not eliminated within the 24-month follow-up period of the previous screening test. Second, the sensitivity and specificity of mammographic screening varied depending on the reader and type of equipment, such as film or digital mammography. Despite inter- and intraradiologist variations, the bias was nondifferential, resulting in a net association with the null hypothesis.^42^ Therefore, the results observed in this study are robust. In addition, the inverse association observed between breast density and IBC might be an artifact of the ultrasound examination; however, we could not control for the use of additional ultrasound examinations because of unavailable information. Finally, when comparing mortality, information on tumor characteristics, stage, and treatment methods for IBC and SBC was not considered.

## Conclusions

In this cohort study, decreased BMI, HRT use after menopause, and alcohol consumption were associated with an increased likelihood of IBC compared with SBC. The lower likelihood of IBC as a breast density category increased and the different associations observed between a family history of breast cancer and IBC by diagnostic period might reflect the wide application of breast ultrasonography for breast cancer screening. Overall mortality of IBC was comparable with that of SBC, but total and cancer-related mortality of IBC diagnosed between 6 and 12 months was higher than that of SBC.

## Notes

Supplement 1. **eTable 1.** Factors Associated With Interval Breast Cancers Compared to Screen-Detected Breast Cancers According to Breast Density **eTable 2.** Factors Associated With Interval Breast Cancers Diagnosed Between 6 and 12 Months After Screening Compared to Screening-Detected Breast Cancer According to Breast Density **eTable 3.** Factors Associated With Interval Breast Cancers Diagnosed Between 12 and 24 Months Compared to Screening-Detected Breast Cancers According to Breast Density

Supplement 2.
Data Sharing Statement
