# Supplementary material for: Risk Factors and Mortality Among Women With Interval Breast Cancer vs Screen-Detected Breast Cancer
Source: JAMA Netw Open. 2024 May 20;7(5):e2411927. doi: 10.1001/jamanetworkopen.2024.11927 (PMC11107304; doi:10.1001/jamanetworkopen.2024.11927)
Supplement: Supplement 1. — eTable 1. Factors Associated With Interval Breast Cancers Compared to Screen-Detected Breast Cancers According to Breast Density eTable 2. Factors Associated With Interval Breast Cancers Diagnosed Between 6 and 12 Months After Screening Compared to Screening-Detected Breast Cancer According to Breast Density eTable 3. Factors Associated With Interval Breast Cancers Diagnosed Between 12 and 24 Months Compared to Screening-Detected Breast Cancers According to Breast Density [file jamanetwopen-e2411927-s001.pdf]

Supplementary Online Content

Song H, Tran TXM, Kim S, Park B. Risk factors and mortality among women with interval breast cancer vs screen-detected breast cancer. *JAMA Netw Open*. 2024;7(5):e2411927. doi:10.1001/jamanetworkopen.2024.11927

eTable 1. Factors Associated With Interval Breast Cancers Compared to Screen-Detected Breast Cancers According to Breast Density

eTable 2. Factors Associated With Interval Breast Cancers Diagnosed Between 6 and 12 Months After Screening Compared to Screening-Detected Breast Cancer According to Breast Density

eTable 3. Factors Associated With Interval Breast Cancers Diagnosed Between 12 and 24 Months Compared to Screening-Detected Breast Cancers According to Breast Density

This supplementary material has been provided by the authors to give readers additional information about their work.

© 2024 Song H et al. *JAMA Network Open.*

eTable 1. Factors Associated With Interval Breast Cancers Compared to Screen-Detected Breast Cancers According to Breast Density

Exposure	Non dense[a]		Dense[a]	
	IBC vs. SBC n = 3,351		IBC vs. SBC n = 5,351	
	cOR (95% CI)	aOR[b] (95% CI)	cOR (95% CI)	aOR[b] (95% CI)
**Age at diagnosis, years**				
40–49	1.46 (1.29–1.66)	0.93 (0.77–1.13)	1.30 (1.15–1.45)	1.01 (0.87–1.18)
50–59	1.17 (1.06–1.30)	1.03 (0.92–1.15)	1.22 (1.09–1.38)	1.11 (0.98–1.26)
60–74	1.00 (reference)	1.00 (reference)	1.00 (reference)	1.00 (reference)
**Body mass index (kg/m^2)**				
Underweight (<18.5)	1.35 (0.84–2.17)	1.27 (0.78–2.05)	1.47 (1.17–1.84)	1.41 (1.12–1.78)
Normal (18.5–23)	1.43 (1.28–1.59)	1.36 (1.21–1.52)	1.24 (1.13–1.36)	1.19 (1.09–1.31)
Overweight (23–25)	1.18 (1.05–1.33)	1.16 (1.04–1.31)	1.22 (1.10–1.36)	1.19 (1.07–1.33)
Obese (≥25)	1.00 (reference)	1.00 (reference)	1.00 (reference)	1.00 (reference)
**Menarche age, years**				
<15	1.27 (1.12–1.44)	1.12 (0.98–1.28)	1.10 (0.99–1.22)	1.00 (0.89–1.12)
15–16	1.18 (1.06–1.32)	1.11 (1.00–1.24)	1.02 (0.92–1.13)	0.94 (0.85–1.05)
≥17	1.00 (reference)	1.00 (reference)	1.00 (reference)	1.00 (reference)
**Menopausal status**				
Pre-menopause	1.56 (1.40–1.74)	1.51 (1.24–1.84)	1.27 (1.17–1.37)	1.08 (0.93–1.25)
Post-menopause	1.00 (reference)	1.00 (reference)	1.00 (reference)	1.00 (reference)
**Oral contraceptives use**				
Never	1.00 (reference)	1.00 (reference)	1.00 (reference)	1.00 (reference)
Ever	0.90 (0.79–1.02)	0.90 (0.79–1.03)	0.97 (0.87–1.08)	0.96 (0.86–1.07)
**Hormone replacement therapy experience**				
Never	1.00 (reference)	1.00 (reference)	1.00 (reference)	1.00 (reference)
Ever	1.33 (1.14–1.55)	1.32 (1.12–1.54)	1.46 (1.24–1.72)	1.45 (1.23–1.72)
**Number of pregnancies**				
None	1.00 (reference)	1.00 (reference)	1.00 (reference)	1.00 (reference)
1 child	1.05 (0.81–1.37)	1.08 (0.81–1.45)	1.03 (0.88–1.21)	0.96 (0.80–1.15)
≥2 children	1.11 (0.89–1.40)	1.24 (0.96–1.61)	1.09 (0.95–1.25)	1.07 (0.91–1.25)
**Breastfeeding experience**				
Never	1.20 (1.04–1.39)	1.03 (0.88–1.19)	1.15 (1.05–1.26)	1.09 (0.99–1.20)
Ever	1.00 (reference)	1.00 (reference)	1.00 (reference)	1.00 (reference)
**Family history of breast cancer**				
No	1.00 (reference)	1.00 (reference)	1.00 (reference)	1.00 (reference)
Yes	0.94 (0.79–1.12)	0.95 (0.79–1.14)	0.83 (0.71–0.96)	0.85 (0.73–0.99)
**Current drinking experience**				
No	1.00 (reference)	1.00 (reference)	1.00 (reference)	1.00 (reference)
Yes	1.19 (1.05–1.34)	1.09 (0.96–1.24)	1.13 (1.04–1.23)	1.10 (1.00–1.20)
**Physical activity**				
No	1.00 (reference)	1.00 (reference)	1.00 (reference)	1.00 (reference)
Yes	0.99 (0.90–1.10)	0.96 (0.86–1.06)	1.08 (0.99–1.18)	1.06 (0.97–1.16)

SBC, screen-detected breast cancer; IBC, interval breast cancer; cOR, crude odds ratio; CI, confidence interval; aOR, multivariate adjusted odds ratio.

[a]Non-dense breast density was defined as BI-RADS breast density categories 1 and 2, and

© 2024 Song H et al. *JAMA Network Open.*

dense breast density was defined as BI-RADS breast density breast density categories 3 and 4.
[b]Multivariate logistic regression model adjusted for age at diagnosis, body mass index, menarche age, menopausal status, oral contraceptive use, hormone replacement therapy experience, number of pregnancies, breastfeeding experience, family history of breast cancer in first-degree relatives, drinking experience, and physical activity.

© 2024 Song H et al. *JAMA Network Open.*

eTable 2. Factors Associated With Interval Breast Cancers Diagnosed Between 6 and 12 Months After Screening Compared to Screening-Detected Breast Cancer According to Breast Density

Exposure	Non dense[a]		Dense[a]	
	IBC 6–12 m vs. SBC n = 820		IBC 6–12 m vs. SBC n = 1,539	
	cOR (95% CI)	aOR[b] (95% CI)	cOR (95% CI)	aOR[b] (95% CI)
**Age at diagnosis, years**				
40–49	2.25 (1.84–2.74)	1.85 (1.37–2.49)	1.71 (1.42–2.07)	1.66 (1.31–2.10)
50–59	1.42 (1.19–1.69)	1.34 (1.11–1.61)	1.36 (1.12–1.65)	1.36 (1.11–1.67)
60–74	1.00 (reference)	1.00 (reference)	1.00 (reference)	1.00 (reference)
**Body mass index (kg/m^2)**				
Underweight (<18.5)	2.13 (1.11–4.08)	1.92 (0.99–3.71)	1.62 (1.16–2.25)	1.51 (1.08–2.12)
Normal (18.5–23)	1.61 (1.35–1.93)	1.48 (1.23–1.77)	1.26 (1.09–1.46)	1.17 (1.01–1.36)
Overweight (23–25)	1.16 (0.96–1.41)	1.13 (0.93–1.38)	1.30 (1.10–1.53)	1.26 (1.07–1.48)
Obese (≥25)	1.00 (reference)	1.00 (reference)	1.00 (reference)	1.00 (reference)
**Menarche age, years**				
<15	1.31 (1.07–1.62)	1.00 (0.80–1.25)	1.06 (0.91–1.24)	0.89 (0.76–1.05)
15–16	1.32 (1.10–1.57)	1.17 (0.97–1.40)	0.91 (0.78–1.06)	0.81 (0.69–0.95)
≥17	1.00 (reference)	1.00 (reference)	1.00 (reference)	1.00 (reference)
**Menopausal status**				
Pre-menopause	1.78 (1.51–2.11)	1.30 (0.94–1.80)	1.28 (1.14–1.44)	0.97 (0.78–1.21)
Post-menopause	1.00 (reference)	1.00 (reference)	1.00 (reference)	1.00 (reference)
**Oral contraceptives use**				
Never	1.00 (reference)	1.00 (reference)	1.00 (reference)	1.00 (reference)
Ever	0.79 (0.63–0.98)	0.83 (0.66–1.04)	1.02 (0.87–1.21)	1.02 (0.86–1.20)
**Hormone replacement therapy experience**				
Never	1.00 (reference)	1.00 (reference)	1.00 (reference)	1.00 (reference)
Ever	1.19 (0.92–1.54)	1.20 (0.92–1.56)	1.52 (1.19–1.94)	1.56 (1.21–2.00)
**Number of pregnancies**				
None	1.00 (reference)	1.00 (reference)	1.00 (reference)	1.00 (reference)
1 child	0.92 (0.61–1.39)	0.89 (0.56–1.39)	1.06 (0.83–1.35)	0.96 (0.74–1.26)
≥2 children	0.93 (0.66–1.32)	1.00 (0.67–1.49)	1.05 (0.85–1.30)	1.01 (0.79–1.29)
**Breastfeeding experience**				
Never	1.31 (1.04–1.63)	1.04 (0.82–1.32)	1.20 (1.05–1.38)	1.12 (0.97–1.28)
Ever	1.00 (reference)	1.00 (reference)	1.00 (reference)	1.00 (reference)
**Family history of breast cancer**				
No	1.00 (reference)	1.00 (reference)	1.00 (reference)	1.00 (reference)
Yes	1.31 (1.01–1.70)	1.31 (1.01–1.70)	1.10 (0.89–1.36)	1.11 (0.90–1.38)
**Current drinking experience**				
No	1.00 (reference)	1.00 (reference)	1.00 (reference)	1.00 (reference)
Yes	1.16 (0.95–1.41)	0.96 (0.78–1.18)	1.20 (1.06–1.37)	1.13 (1.00–1.29)
**Physical activity**				
No	1.00 (reference)	1.00 (reference)	1.00 (reference)	1.00 (reference)
Yes	1.04 (0.88–1.23)	1.00 (0.84–1.19)	1.19 (1.05–1.36)	1.16 (1.02–1.33)

SBC, screen-detected breast cancer; IBC, interval breast cancer; cOR, crude odds ratio; CI, confidence interval; aOR, multivariate adjusted odds ratio.

© 2024 Song H et al. *JAMA Network Open.*

[a]Non-dense breast density was defined as BI-RADS breast density categories 1 and 2, and dense breast density was defined as BI-RADS breast density breast density categories 3 and 4.
[b]Multivariate logistic regression model adjusted for age at diagnosis, body mass index, menarche age, menopausal status, oral contraceptive use, hormone replacement therapy experience, number of pregnancies, breastfeeding experience, family history of breast cancer in first-degree relatives, drinking experience, and physical activity.

© 2024 Song H et al. *JAMA Network Open.*

eTable 3. Factors Associated With Interval Breast Cancers Diagnosed Between 12 and 24 Months Compared to Screening-Detected Breast Cancers According to Breast Density

Exposure	Non dense[a]		Dense[a]	
	IBC 12–24 m vs. SBC n = 2,531		IBC 12–24 m vs. SBC n = 3,812	
	cOR (95% CI)	aOR[b] (95% CI)	cOR (95% CI)	aOR[b] (95% CI)
**Age at diagnosis, years**				
40–49	1.26 (1.09–1.45)	0.74 (0.60–0.91)	1.16 (1.03–1.32)	0.83 (0.71–0.98)
50–59	1.11 (0.99–1.24)	0.95 (0.84–1.07)	1.18 (1.04–1.34)	1.02 (0.89–1.18)
60–74	1.00 (reference)	1.00 (reference)	1.00 (reference)	1.00 (reference)
**Body mass index (kg/m^2)**				
Underweight (<18.5)	1.11 (0.64–1.90)	1.06 (0.61–1.83)	1.41 (1.09–1.81)	1.37 (1.06–1.77)
Normal (18.5–23)	1.37 (1.21–1.54)	1.32 (1.17–1.49)	1.24 (1.12–1.37)	1.20 (1.08–1.33)
Overweight (23–25)	1.19 (1.05–1.35)	1.18 (1.04–1.33)	1.19 (1.06–1.34)	1.17 (1.04–1.32)
Obese (≥25)	1.00 (reference)	1.00 (reference)	1.00 (reference)	1.00 (reference)
**Menarche age, years**				
<15	1.26 (1.10–1.44)	1.16 (1.00–1.34)	1.12 (1.00–1.26)	1.05 (0.93–1.19)
15–16	1.14 (1.01–1.28)	1.09 (0.97–1.23)	1.06 (0.95–1.19)	1.01 (0.90–1.13)
≥17	1.00 (reference)	1.00 (reference)	1.00 (reference)	1.00 (reference)
**Menopausal status**				
Pre-menopause	1.49 (1.33–1.68)	1.59 (1.28–1.96)	1.26 (1.16–1.37)	1.13 (0.96–1.33)
Post-menopause	1.00 (reference)	1.00 (reference)	1.00 (reference)	1.00 (reference)
**Oral contraceptives use**				
Never	1.00 (reference)	1.00 (reference)	1.00 (reference)	1.00 (reference)
Ever	0.93 (0.81–1.07)	0.92 (0.80–1.06)	0.95 (0.84–1.07)	0.94 (0.83–1.07)
**Hormone replacement therapy experience**				
Never	1.00 (reference)	1.00 (reference)	1.00 (reference)	1.00 (reference)
Ever	1.37 (1.16–1.62)	1.35 (1.14–1.60)	1.44 (1.20–1.73)	1.41 (1.18–1.69)
**Number of pregnancies**				
None	1.00 (reference)	1.00 (reference)	1.00 (reference)	1.00 (reference)
1 child	1.11 (0.83–1.48)	1.16 (0.84–1.60)	1.02 (0.85–1.22)	0.97 (0.79–1.18)
≥2 children	1.19 (0.92–1.52)	1.34 (1.00–1.78)	1.11 (0.95–1.29)	1.09 (0.91–1.30)
**Breastfeeding experience**				
Never	1.17 (1.00–1.37)	1.03 (0.88–1.22)	1.12 (1.02–1.24)	1.08 (0.97–1.20)
Ever	1.00 (reference)	1.00 (reference)	1.00 (reference)	1.00 (reference)
**Family history of breast cancer**				
No	1.00 (reference)	1.00 (reference)	1.00 (reference)	1.00 (reference)
Yes	0.82 (0.68–1.01)	0.83 (0.68–1.02)	0.72 (0.60–0.86)	0.74 (0.62–0.88)
**Current drinking experience**				
No	1.00 (reference)	1.00 (reference)	1.00 (reference)	1.00 (reference)
Yes	1.20 (1.05–1.36)	1.14 (0.99–1.30)	1.10 (1.00–1.21)	1.08 (0.98–1.20)
**Physical activity**				
No	1.00 (reference)	1.00 (reference)	1.00 (reference)	1.00 (reference)
Yes	0.98 (0.88-1.10)	0.94 (0.84–1.05)	1.04 (0.95–1.14)	1.02 (0.93–1.12)

SBC, screen-detected breast cancer; IBC, interval breast cancer; cOR, crude odds ratio; CI, confidence interval; aOR, multivariate adjusted odds ratio.

© 2024 Song H et al. *JAMA Network Open.*

[a]Non-dense breast density was defined as BI-RADS breast density categories 1 and 2, and dense breast density was defined as BI-RADS breast density breast density categories 3 and 4.
[b]Multivariate logistic regression model adjusted for age at diagnosis, body mass index, menarche age, menopausal status, oral contraceptive use, hormone replacement therapy experience, number of pregnancies, breastfeeding experience, family history of breast cancer in first-degree relatives, drinking experience, and physical activity.

© 2024 Song H et al. *JAMA Network Open.*
