# Supplementary material for: Risk Factors and Mortality Among Women With Interval Breast Cancer vs Screen-Detected Breast Cancer
Source: JAMA Netw Open. 2024 May 20;7(5):e2411927. doi: 10.1001/jamanetworkopen.2024.11927 (PMC11107304; doi:10.1001/jamanetworkopen.2024.11927)
Supplement: Supplement 2. — Data Sharing Statement [file jamanetwopen-e2411927-s002.pdf]

Data Sharing Statement

Song. Risk Factors and Mortality Among Women With Interval Breast Cancer vs Screen-Detected Breast Cancer. *JAMA Netw Open*. Published May 20, 2024.
doi:10.1001/jamanetworkopen.2024.11927

Data
Data available: No

Additional Information
Explanation for why data not available: The data used in this study were obtained from the Korean National Health Information Database. Data sharing determined by the Korean National Health Information Database and not by the authors.
